# Testosterone Treatment, Weight Loss, and Health-related Quality of Life and Psychosocial Function in Men: A 2-year Randomized Controlled Trial

**DOI:** 10.1210/clinem/dgae085

**Published:** 2024-02-09

**Authors:** Mathis Grossmann, Kristy P Robledo, Mark Daniel, David J Handelsman, Warrick J Inder, Bronwyn G A Stuckey, Bu B Yeap, Mark Ng Tang Fui, Karen Bracken, Carolyn A Allan, David Jesudason, Jeffrey D Zajac, Gary A Wittert

**Affiliations:** Department of Medicine, Austin Health, The University of Melbourne, Heidelberg, VIC 3084, Australia; Department of Endocrinology, Austin Health, Heidelberg, VIC 3084, Australia; NHMRC Clinical Trials Centre, University of Sydney, Camperdown, NSW 2050, Australia; Department of Population Health, Dasman Diabetes Institute, Kuwaiit City, Kuwait; Department of Andrology, ANZAC Research Institute, University of Sydney and Department of Andrology, Concord Hospital, Concord, NSW 2139, Australia; Department of Endocrinology, Princess Alexandra Hospital and the University of Queensland, Woolloongabba, QLD 4102, Australia; Keogh Institute for Medical Research, Department of Endocrinology and Diabetes, Sir Charles Gairdner Hospital and Medical School, University of Western Australia, Western Australia, Woolloongabba, QLD 4102, Australia; Medical School, University of Western Australia and Department of Endocrinology and Diabetes, Fiona Stanley Hospital, Nedlands, WA 6009, Australia; Department of Medicine, Austin Health, The University of Melbourne, Heidelberg, VIC 3084, Australia; Department of Endocrinology, Austin Health, Heidelberg, VIC 3084, Australia; NHMRC Clinical Trials Centre, University of Sydney, Camperdown, NSW 2050, Australia; School of Clinical Sciences at Monash Health and Faculty of Medicine, Nursing and Health Sciences, Centre for Endocrinology and Metabolism, Hudson Institute of Medical Research, Monash University, Clayton, VIC 3168, Australia; Department of Medicine, University of Adelaide, Adelaide, SA 5005, Australia; Department of Endocrinology, The Queen Elizabeth Hospital, Woodville South, SA 5011, Australia; Department of Medicine, Austin Health, The University of Melbourne, Heidelberg, VIC 3084, Australia; Department of Endocrinology, Austin Health, Heidelberg, VIC 3084, Australia; Freemasons Foundation Centre for Men's Health, University of Adelaide, Adelaide, SA 5001, Australia

**Keywords:** testosterone, psychosocial health, quality of life, T4DM

## Abstract

**Objective:**

To determine the effect of testosterone vs placebo treatment on health-related quality of life (HR-QOL) and psychosocial function in men without pathologic hypogonadism in the context of a lifestyle intervention.

**Design, Setting, Participants:**

Secondary analysis of a 2-year randomized controlled testosterone therapy trial for prevention or reversal of newly diagnosed type 2 diabetes, enrolling men ≥ 50 years at high risk for type 2 diabetes from 6 Australian centers.

**Interventions:**

Injectable testosterone undecanoate or matching placebo on the background of a community-based lifestyle program.

**Main Outcomes:**

Self-reported measures of HR-QOL/psychosocial function.

**Results:**

Of 1007 participants randomized into the Testosterone for Type 2 Diabetes Mellitus (T4DM) trial, 648 (64%) had complete data available for all HR-QOL/psychosocial function assessments at baseline and 2 years. Over 24 months, while most measures were not different between treatment arms, testosterone treatment, compared with placebo, improved subjective social status and sense of coherence. Baseline HR-QOL/psychosocial function measures did not predict the effect of testosterone treatment on glycemic outcomes, primary endpoints of T4DM. Irrespective of treatment allocation, larger decreases in body weight were associated with improved mental quality of life, mastery, and subjective social status. Men with better baseline physical function, greater sense of coherence, and fewer depressive symptoms experienced greater associated decreases in body weight, with similar effects on waist circumference.

**Conclusion:**

In this diabetes prevention trial, weight loss induced by a lifestyle intervention improved HR-QOL and psychosocial function in more domains than testosterone treatment. The magnitude of weight and waist circumference reduction were predicted by baseline physical function, depressive symptomology, and sense of coherence.

In men with classical hypogonadism due to organic hypothalamic-pituitary or testicular pathology, uncontrolled (for ethical reasons) observational studies suggest that testosterone replacement improves self-reported sense of energy and mood (1, 2). In men without organic hypogonadism, the effects of testosterone treatment on health-related quality of life (HR-QOL) and psychosocial function outcomes are less clear but require efficacy and safety evidence. In the T-Trials (3), an integrated series of randomized controlled trials (RCTs) that recruited 780 US men aged 65 years or older with a serum testosterone concentration of <9.4 mmol/L (<275 ng/dL), topical testosterone treatment over 12 months, compared with placebo, did not increase the primary outcome measure of vitality in the subgroup of men (n = 474) with self-reported low vitality [12-item Short Form Survey (SF-12) vitality subscale]. Testosterone treatment in these men did, however, predict small but statistically significant effects on mood (positive and negative affect scale) and depressive symptoms (Patient Health Questionnaire-9) (3). A meta-analysis of 4 RCTs (including the T-Trials) concluded, on the other hand, that testosterone treatment had no effect on energy or mood (4). In a 2023 individual patient data meta-analysis, testosterone relative to placebo significantly improved the Aging Males’ Symptoms score and 3 of the 10 subscores of the 36-item Short Form Survey, namely social functioning, role limitations due to emotional problems, and the mental health composite score; however, there was no effect on psychological symptoms using the Beck Depression Inventory (5). Further, this meta-analysis did not quantify testosterone effects relating to the underlying standard of care, lifestyle background. A recent analysis of TRAVERSE, a large cardiovascular outcome study, used the patient Health Questionnaire 9 score, the 15-item Geriatric depression scale , and the mood domain of Hypogonadism Impact of Symptoms Questionnaire to assess the effects of testosterone treatment on mood and depressive symptoms (6). Testosterone, compared to placebo, had no effect on depressive symptoms in the small (1.5%) subgroup of men with late-life onset, low-grade persistent depressive disorders (n = 49). In contrast, in men with minor or more depressive symptoms (n = 2643) and in all participants (n = 5024), testosterone treatment was associated with small but significant improvements in mood and energy but not in cognition or sleep quality (6). Finally in TEAAM, a study designed to assess the effects of testosterone on atherosclerosis progression, testosterone therapy over 3 years had no effect on HR-QOL using the 36-item Short Form Survey either on the composite score or on any of the subdomains (7). None of these trials, in contrast to the Testosterone for Type 2 Diabetes Mellitus (T4DM) trial, included a lifestyle program. Other literature from largely observational studies, often based on salivary testosterone concentration, suggests that endogenous serum testosterone may drive the motivation to achieve or maintain a high social status, increase motivational drive and stress resilience, and reduce fearfulness (8-10).

The T4DM trial was a 2-year RCT enrolling 1007 men aged 50 to 74 years at high risk of type 2 diabetes (T2D) and with a serum testosterone (by screening immunoassay) of <14 nmol/L. Its main analysis reported that injectable testosterone treatment over 24 months, together with a lifestyle improvement program, reduced the risk of T2D at study end by 40% [relative risk 0.59, 95% confidence interval (CI) .43-.80; *P* = .0007] but without a significant effect on reducing hemoglobin A1c (11).

This current analysis of T4DM data interrogated the effect of testosterone treatment on measures of HR-QOL and psychosocial function, seeking to determine the relationships between psychosocial health and the influence of testosterone treatment on glycemic outcomes. We tested 2 prespecified hypotheses (12): first, that HR-QOL and psychosocial function (including subjective social status, depressive symptomology, mastery, and sense of coherence) will improve over time and be greater at study end in men receiving testosterone compared with placebo, and second, that HR-QOL and psychosocial function will moderate the effect of testosterone treatment on glycemic outcomes. Finally, we tested the 2 post hoc hypotheses that changes in body weight will moderate the effect of testosterone on HR-QOL and psychosocial function and that baseline HR-QOL and psychosocial function will predict body weight outcomes at study end in the context of a lifestyle intervention program common to both arms of the trial.

## Methods

### Data Source

The T4DM protocol (12) and efficacy results (11) are published. Briefly, T4DM was a multisite Australian randomized, double-blind, placebo-controlled, 2-year phase 3b trial designed to evaluate the effects of testosterone treatment on the background of a lifestyle intervention program on diabetes risk, as assessed by 2 primary outcomes: (1) 2-hour glucose ≥ 11.1 mmol/L and (2) the change in 2-hour glucose from baseline, both as measured by the oral glucose tolerance test (OGTT) at 2 years. To be eligible, participants had to be male and aged 50 to 74 years with a waist circumference ≥95 cm, have impaired glucose tolerance or newly diagnosed T2D, and have a fasting serum testosterone drawn between 8 and 10 Am of ≤14 nmol/L by immunoassay at an accredited pathology provider (Sonic Health Care, Australia). Men were selected based on a serum testosterone of ≤14 nmol/L as previous work in Australian men has shown that this is the testosterone threshold below which the risk of T2D (the primary endpoint of the study) increases substantially (13). Exclusion criteria included organic hypothalamic-pituitary-testicular pathology, testosterone treatment in the past 12 months, or history of androgen abuse at any time. All 1007 participants were given access to a lifestyle program (WW, formerly Weight Watchers) and randomized (1:1) to testosterone undecanoate (1000 mg) or matched placebo, both administered by clinic staff via deep intramuscular injection every 3 months for 2 years. The study received ethics committee approval to be conducted at each site and was registered on the Australia and New Zealand Clinical Trials Registry (ACTRN12612000287831).

### Questionnaires

To assess HR-QOL and psychosocial function, the following 5 validated questionnaires were used: the SF-12 (HR-QOL) and psychosocial instruments examining different aspects of psychosocial function including the MacArthur Scale of Subjective Social Status (MAC), Pearlin Personal Mastery Scale (Mastery) and Sense of Coherence (SOC), and the Center for Epidemiological Studies-Depression (CES-D) questionnaire. T4DM trial participants completed the CES-D at baseline, week 54, and week 104 only, while the other 4 questionnaires were completed at baseline and weeks 30, 54, 78, and 104 (study end).

Physical and mental function was assessed using the SF-12, a 12-item questionnaire that assesses both physical and mental domains of HR-QOL, ie, SF-12 physical and SF-12 mental, analyzed separately as described (14).

The MAC (15) is a 2-item questionnaire designed to assess subjective social status (ie, a person's belief about their location in a status order). Using a Likert scale of 1 to 10, participants rate their social status or standing, with 10 representing those who are best off (ie, they have the most money, the most education, and the most respected jobs) and 1 representing the people worst off (ie, who have the least money, the least education, and the least respected jobs or no jobs). In MAC1, participants are asked to rank themselves “relative to people like yourself” and in MAC2 “within society generally.”

The Mastery scale (16) is a 7-item questionnaire designed to assess the extent to which people feel they are in control of the forces affecting their lives. Using a 4-point Likert scale (from 1, “strongly disagree” to 4, “strongly agree”), the Mastery scale measures the extent to which an individual regards their life chances as being under their personal control rather than fatalistically ruled. Scores range from 4 to 28, with higher numbers indicating a stronger sense of control and, thus, presumably stronger capacity for coping with perceived adversities. Respondents rated their perceived mastery for the 4 weeks prior to completing the questionnaire.

The SOC (17) is a 13-item questionnaire designed to assess, over the last 4 weeks, how individuals view their life and how they use their resistance resources to maintain and develop their health. It uses a 7-point Likert scale with response options relevant to each item. Scores range from 7 to 91. Higher scores indicate greater levels of sense of coherence.

The CES-D questionnaire is a 20-item measure that asks individuals to rate how often over the past week they experienced depressive symptomology, such as restless sleep, poor appetite, and feeling lonely. Response options range from 0 to 3 for each item (0 = rarely or none of the time, 1 = some or little of the time, 2 = moderately or much of the time, 3 = most or almost all the time). Scores range from 0 to 60, with high scores indicating greater depressive symptoms (18).

### Statistical Analyses

These secondary, prespecified analyses were performed by intention to treat, using all available data up until the completion of the trial at 2 years. Analyses were performed in the R computing environment, and no adjustments have been made for multiple comparisons. *P* less than .05 was considered statistically significant.

Baseline characteristics were compared for participants with data available vs those without. First, HR-QOL and psychosocial function was assessed for change over time and whether changes were consistent across treatment arms. Each HR-QOL outcome (SF-12 physical and mental) and psychosocial function parameter (MAC1, MAC2, Mastery, SOC, and CES-D) was modeled over time using a generalized linear model, adjusting for visit, treatment group, and the relevant baseline HR-QOL and psychosocial function parameter, taking into account the correlation within each subject over time. An interaction term was fitted to assess whether trends over time varied by treatment group (treatment by visit interaction). If the interaction was *P* > .05, the interaction was dropped from subsequent models. A baseline testosterone by LCMS term was also included in these models to assess if the effect of HR-QOL/psychosocial function was impacted by baseline testosterone concentrations.

Baseline psychosocial function for MAC1, MAC2, Mastery, and SOC was assessed to distinguish participants who experienced a more (or less) favorable effect of treatment on 2 outcomes of glucose. For glucose measured by OGTT at 2 years, a linear model was fitted adjusting for baseline glucose by OGTT, baseline HR-QOL/psychosocial function, treatment, and the interaction between treatment and baseline HR-QOL/psychosocial function. Fasting plasma glucose was measured at more than 1 timepoint; therefore, a generalized linear model was fitted, adjusting for baseline fasting plasma glucose, visit, treatment, and the relevant baseline HR-QOL/psychosocial function, accounting for the correlation within each subject over time.

Associations between baseline HR-QOL/psychosocial function and body composition at 2 years were assessed. For weight and waist circumference, linear models were graphed according to whether or not participants were attending the WW program (either online or face to face) at each visit.

Lastly, the impact of WW program attendance over time was also assessed for each HR-QOL/psychosocial function measure in generalized linear models.

## Results

Of the 1007 participants randomized into T4DM, 648 (64%) had complete data available for all HR-QOL and psychosocial function assessments at baseline and 2 years. Those with data and those without data were generally well matched in terms of baseline characteristics, except those with data were more likely to be married, report fewer depressive symptoms, be more highly educated, and have higher incomes (Supplementary Table S1) (19). Baseline characteristics for the 648 men with complete HR-QOL and psychosocial data are given in Table 1.

**Table 1.** Comparison of baseline characteristics between treatment arms for those with complete data*^a^* available

Characteristic	Placebo, n = 319	Testosterone, n = 329	*P*-value*^b^*
Age at baseline (years), mean (SD)	60 (7)	60 (6)	.20
60 years or older, n (%)	167 (52)	150 (46)	.10
Current smoker, n (%)	12 (3.8)	16 (4.9)	.62
Waist circumference (cm), mean (SD)	118 (11)	117 (12)	.46
Waist circumference groups, n (%)			.75
=<100 cm	15 (4.7)	17 (5.2)	
101-115 cm	120 (38)	132 (40)	
>115 cm	184 (58)	180 (55)	
Weight (kg), mean (SD)	108 (17)	107 (17)	.28
BMI categories, n (%)			.46
Normal (18.50-24.99 kg/m²)	2 (0.6)	3 (0.9)	
Overweight (25.00-29.99 kg/m²)	57 (18)	59 (18)	
Obese (30.00-34.99 kg/m²)	117 (37)	129 (39)	
Severely obese (35.00-39.99 kg/m²)	89 (28)	99 (30)	
Very severely obese (>=40 kg/m²)	54 (17)	39 (12)	
History of T2D, n (%)	126 (39)	144 (44)	.31
History of prostate cancer, n (%)	41 (13)	33 (10)	.31
SSRI use at baseline, n (%)	17 (5.3)	22 (6.7)	.57
Baseline testosterone (nmol/L), mean (SD)	13.9 (4.7)	13.6 (4.1)	.41
Unknown	15	17	
Baseline testosterone groups, n (%)			.038
Low (<8.0 nmol/L)	28 (9.2)	16 (5.1)	
Medium (8.0 to <11.0 nmol/L)	55 (18)	76 (24)	
High (>=11.0 nmol/L)	221 (73)	220 (71)	
Unknown	15	17	
Baseline glucose groups, n (%)			.42
Normal (<7.8 mmol/L)*	1 (0.3)	4 (1.2)	
Prediabetes (=7.8 to <11.1 mmol/L)	254 (80)	260 (79)	
Type 2 diabetes (=11.1 to <15 mmol/L)	64 (20)	65 (20)	
Country of birth, n (%)			.92
Asia	7 (2.2)	9 (2.7)	
Australia/New Zealand	234 (73)	236 (72)	
Europe	48 (15)	49 (15)	
Other	30 (9.4)	35 (11)	
FPG, mean (SD)	6.06 (0.88)	6.09 (0.89)	.65
CES-D score, mean (SD)	4.5 (4.0)	5.2 (4.4)	.047
Unknown	25	28	
Married, n (%)	280 (88)	292 (89)	.79
Annual income, n (%)			.089
$100 001-$150 000	62 (20)	88 (28)	
$50 001-$100 000	111 (37)	96 (30)	
<$50 000	53 (17)	45 (14)	
>$150 001	77 (25)	86 (27)	
Unknown	16	14	
Employment, n (%)			.91
Employed for wages	168 (53)	172 (52)	
Retired/unemployed	95 (30)	95 (29)	
Self-employed/family business	56 (18)	62 (19)	
Education level, n (%)			.043
Diploma	75 (24)	71 (22)	
School certificate or lower	74 (23)	75 (23)	
Trade	68 (22)	48 (15)	
University or higher	99 (31)	132 (40)	
Unknown	3	3	

Abbreviations: BMI, body mass index; CES-D, Center for Epidemiological Studies-Depression; FPG, fasting plasma glucose; SSRI, selective serotonin reuptake inhibitor; T2D, type 2 diabetes.

^*a*^Complete data was defined on a per patient basis as all health-related quality of life measures available at baseline and 2 years.

^*b*^Two-sample t-test; Chi-squared test.

### Adjusted Effects of Testosterone Treatment on HR-QoL and Psychosocial Measures

**Figure 1.**
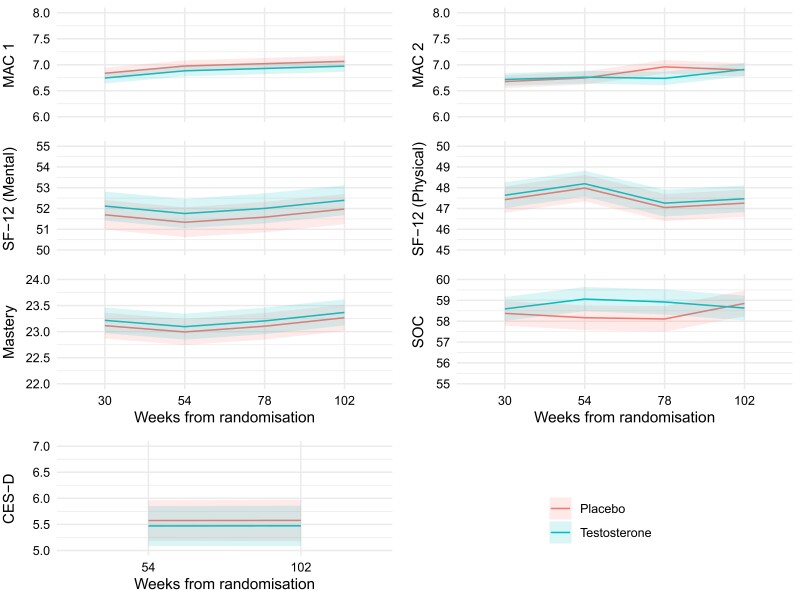
Effect of testosterone treatment on health-related quality of life and psychosocial function measures. Shown are the effects of testosterone and placebo treatment on on health-related quality of life and psychosocial function measures. Data are shown as mean and 95% confidence intervals.

Of the 1007 participants randomized, the participants included in each model totaled 853 for Mastery, 912 for CES-D, 932 for SF-12 mental/physical and SOC, 935 for MAC2, and 937 for MAC1. In testosterone compared with placebo-treated men, there were no differences over time in SF-12 mental and physical scores, MAC2, Mastery, or the CES-D (all *P*-interaction > .20, Table 2). In contrast, there was evidence of a treatment by time interaction for MAC1 and SOC (*P*-interaction = .04 and .03, respectively). For MAC1, the largest treatment effect was found at around 78 weeks with an increase in MAC1 scores with testosterone treatment, indicative of an improvement with testosterone treatment (*P*-interaction = .04). For SOC, the largest difference was at 54 to 78 weeks, indicative of an improvement with testosterone treatment (*P*-interaction = .03, Fig. 1). The size of the effects of treatment did not depend on the baseline quality of life measurement (all interaction *P* > .31, Supplementary Table S2) (19). Baseline testosterone concentrations had no association with HR-QOL and psychosocial functions, except for the SF-12 mental instrument. For each 1 nmol/L increase in baseline testosterone, SF-12 mental scores were reduced on average by 0.13 units (95%CI: .03-.23, *P* = .011, Supplementary Table S3) (19).

**Table 2.** Interactions between treatment and time for each HR-QOL outcome

Outcome	Treatment by time interaction *P*-value*^a^*
SF-12 (physical)	.79
SF-12 (mental)	.88
MAC1	.04
MAC2	.23
Mastery	.20
SOC	.03
CES-D	.22

Abbreviations: CES-D, Center for Epidemiological Studies-Depression; HR-QOL, health-related quality of life; MAC1/2, Mental, Mastery, and Subjective Social Status; SF-12, 12-item Short Form Survey; SOC, Sense of Coherence.

^*a*^In a generalized linear model for each quality of life outcome, adjusted for visit, treatment, the relevant baseline HR-QOL and time by treatment interaction, taking into account the correlation between measurements taken in the same participant over time.

### Assessing Moderation of Glucose Effects by Baseline HR-QOL and Psychosocial Function Measures

For both outcomes of glucose (fasting plasma glucose and 2-hour glucose by OGTT), there was no evidence of an interaction between any of the baseline HR-QOL and psychosocial measures and treatment. This indicates that the effect of testosterone treatment on measures of glycemia at 2 years was not influenced by a participant’s baseline HR-QOL or psychosocial status; that is, the treatment effect was consistent over all baseline HR-QOL and psychosocial measures (Supplementary Table S4) (19).

### Baseline HR-QOL and Psychosocial Function Measures Predicting Weight, Waist Circumference, and WW Attendance at 2 Years

Table 3 shows the association of baseline QOL and each of the psychosocial measures with body composition outcomes among the study participants. Baseline SF-12 physical, SOC, and depressive symptomology all had associations with weight at 2 years, while Mastery, SF-12 mental, and MAC1 and MAC2 did not. Only the baseline of the SF-12 physical measure and depressive symptomology were associated with waist circumference.

**Table 3.** Effects of baseline HR-QOL on body composition outcomes at 2 years

	Weight at 2 years		Waist circumference at 2 years		Attendance at WW	
Baseline QOL	Beta (95% CI)	*P*-value	Beta (95% CI)	*P*-value	Odds ratio (95% CI)	*P*-value
SF-12 (physical)	−.05 (−.09 to .00)	.042	−.05 (−.10 to −.01)	.016	.99 (.97 to 1.00)	.15
SF-12 (mental)	−.04 (−.08 to .01)	.12	−.03 (−.07 to .01)	.16	1.03 (1.01 to 1.05)	.002
SOC total	−.05 (−.10 to −.01)	.028	−.04 (−.09 to .00)	.073	1.00 (.99 to 1.02)	.62
Mastery	−.12 (−.26 to .01)	.078	−.12 (−.25 to .01)	.060	1.00 (.96 to 1.05)	.89
MAC1	−.10 (−.36 to .16)	.45	−.15 (−.40 to .10)	.23	.99 (.90 to 1.08)	.76
MAC2	−.12 (−.38 to .13)	.35	−.18 (−.42 to .06)	.15	.98 (.89 to 1.07)	.63
Depression	.11 (.01 to .20)	.027	.11 (.02 to .20)	.016	.97 (.94 to 1.00)	.060

Abbreviations: CI, confidence interval; HR-QOL, health-related quality of life; MAC1/2, Mental, Mastery, and Subjective Social Status; QOL, quality of life; SF-12, 12-item Short Form Survey; SOC, Sense of Coherence.

A 1-unit increase in the baseline SF-12 and SOC scores was associated with an average decrease in weight of 0.05 kg (95% CI: .09-.00) and 0.05 kg (95% CI: −.10 to −.01), respectively. For depressive symptomology, a 1-unit increase in the CES-D score was associated with an average increase in weight of 0.11 kg (95% CI: .01-.20). Similar relationships with waist circumference at 2 years were associated with the baseline SF-12 physical and CES-D scores.

A total of 28% (183/648) of participants were attending WW after 2 years. In assessing predictors of WW attendance at 2 years, a sole relationship was found with the SF-12 mental score. For a 1-unit increase in the baseline SF-12 mental score, patients had on average a 3% higher odds of attending WW (95% CI: 1.01-1.05) compared with not attending WW.

### Assessing the Effect of Changes in Weight During the Study

**Figure 2.**
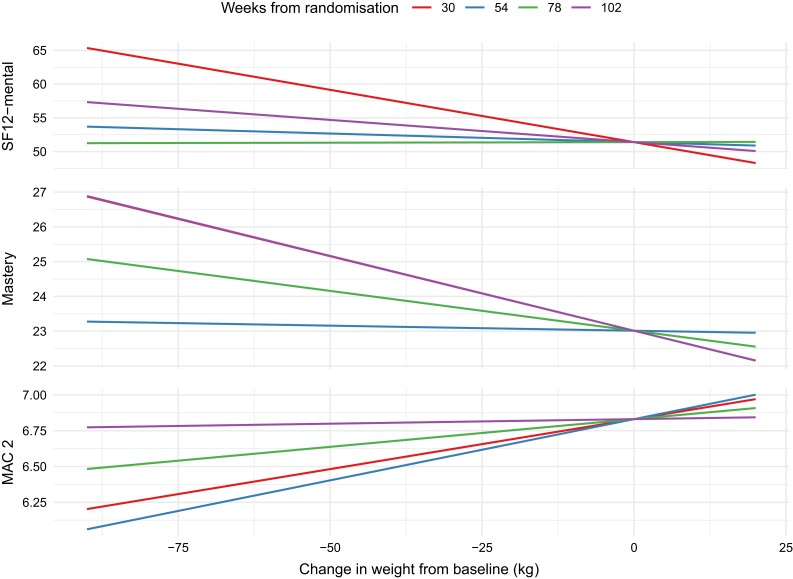
Effects of changes in weight on SF-12 MAC2. Shown are effects of changes in weight during the 2-year trial from baseline on SF-12 MAC2 scores at different weeks from randomization. Abbreviations: MAC2, Mental, Mastery, and Subjective Social Status; SF-12, 12-item Short Form Survey.

The effect of treatment (testosterone or placebo) on HR-QOL and psychosocial outcomes was adjusted for changes in weight over the trial period (visit by change in weight interaction). There was no evidence of a 3-way interaction between treatment, visit, and change in weight (all *P*-interaction > .12). Significant interactions were evident for SF-12 (mental) (*P*-interaction <.001), MAC1 (*P*-interaction = .003), and Mastery (*P*-interaction = .02), while *P*-values for interactions for other HR-QOL and psychosocial scores were >0.46. For SF-12 mental, this interaction (Fig. 2A) showed that large decreases in weight as early as 6 months into the study were associated with greater improvement in mental health scores (eg, 25 kg weight loss corresponds to average SF-12 score of 55) as compared with more moderate changes in weight (eg, 10 kg weight loss corresponds to average SF-12 score of 52). Large changes in weight at 2 years, however, corresponded to a relatively lower improvement in SF-12 mental score (eg, 25 kg weight loss corresponds to average SF-12 score of 53) compared with the same weight change at 6 months, while at 12 and 18 months, changes in weight on average had very little impact on SF-12 mental scores. For Mastery, this interaction showed that large decreases in weight were associated with, on average, higher scores for Mastery for 6 months and 2 years (eg,. both show 25 kg weight loss corresponds to average Mastery score of 24). At 12 and 18 months, changes in weight had little impact on Mastery scores, on average (Fig. 2B). For MAC1, changes in weight at 2 years had little impact on average scores; however, large effects were seen at early visits for those men who lost weight (eg. weight loss of 25 kg corresponds to average MAC1 score of 6.7 at 6 months, compared someone with no change in weight, Fig. 2C).

### Assessing the Effect of Changes in Waist Circumference During the Study

**Figure 3.**
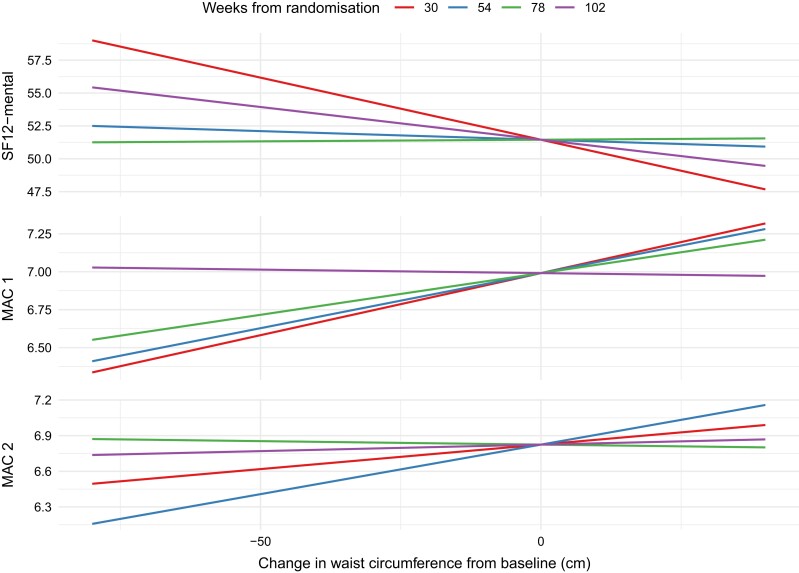
Effects of changes in waist circumference on SF-12 MAC1 and MAC2. Shown are effects of changes in waist circumference during the 2-year trial from baseline on SF-12 MAC1 and MAC2 scores at different weeks from randomisation. Abbreviations: MAC1/2, Mental, Mastery, and Subjective Social Status; SF-12, 12-item Short Form Survey.

The effect of treatment (testosterone or placebo) on HR-QOL and each psychosocial outcome was adjusted for changes in waist circumference over the trial period (visit by change in waist circumference interaction). There was no evidence of a 3-way interaction between treatment, visit, and change in waist circumference (all *P*-interaction > .20). Interactions were evident for SF-12 (mental) (*P*-interaction = .04), MAC1 (*P*-interaction = .02), and MAC2 (*P*-interaction = .03) while *P*-interactions were >.21 for other HR-QOL and psychosocial scores (Fig. 3).

### Assessing the Impact of Lifestyle Adherence on HR-QOL and Psychosocial Outcomes

There was no impact of lifestyle adherence over time on MAC1, MAC2, SOC, or SF-12 physical (*P* > .20). For SF-12-mental, WW attendance was associated with higher scores (1.2, 95% CI:.5-1.9, *P* < .001), while for depression WW attendance was associated with lower scores (0.63, 95% CI: .08-1.2, *P* = .024, Supplementary Table S5) (19). For Mastery, WW was associated with a small increase (0.22, 95% CI: −.01-.44, *P* = .058).

## Discussion

This was a detailed secondary analysis of T4DM, a trial to assess the effects of 2 years of testosterone treatment compared with placebo on incident diabetes on the background of a lifestyle program administered by WW in men at high risk or with recently diagnosed T2D. This current analysis focused on the effects of testosterone treatment on a comprehensive set of self-reported HR-QOL and psychosocial measures using established questionnaires. The main findings are as follows: first, testosterone treatment compared with placebo had no consistent effects on HR-QOL measures or psychosocial factors. However, testosterone treatment improved sense of coherence evident around week 54 to week 78 of the trial, as well as temporarily increased subjective social status around week 78, but had no effect on mental and physical function (SF-12) or depressive symptoms (CES-D). Second, baseline HR-QOL measures did not modify the effects of testosterone treatment on glucose outcomes of T4DM, ie, 2-hour OGTT glucose and the change in 2-hour glucose from baseline. Third, irrespective of treatment status (testosterone or placebo), improvement in mental well-being was associated with changes in body weight during the RCT, meaning that larger decreases in body weight were associated with better self-reported mental quality of life (as assessed by the SF-12 mental health scale). This effect was particularly evident early, ie, over the first 6 months of the testosterone treatment, but also evident at study end (24 months). Fourth, baseline HR-QOL of life and psychosocial measures, independently of treatment, predicted changes in body weight. Specifically, men with better self-reported baseline physical function, greater sense of coherence, and less depressive symptomology had greater associated decreases in body weight. Similar effects were seen for waist circumference. Fifth, those men with higher baseline self-reported SF-12 mental scores had higher attendance at WW at 24 months, ie, study end. Lastly, WW attendance did not impact subjective social status, sense of coherence, or SF-12 physical function scores. WW attendance, however, was associated with higher SF-12 mental scores and lower depressive symptoms (CED-S) and with a small increase in Mastery.

The unique feature of the T4DM study design, ie, randomization to testosterone or placebo treatment on the background of a lifestyle program, the latter administered to all participants, allowed us to assess the effects of a lifestyle intervention with or without testosterone treatment. The analyses conducted here lend support for prioritizing weight loss induced by lifestyle change in the context of exposure to a lifestyle program—rather than testosterone therapy per se—for improving HR-QOL and psychosocial functioning. Testosterone therapy, consistent with a previous meta-analysis (4), did not, with the exception of sense of coherence and subjective social status, improve HR-QOL or other psychosocial measures compared with placebo as assessed by multiple questionnaires in this large 2-year RCT. Interestingly, there was an apparent time-limited effect of testosterone on sense of coherence and subjective social status that did not persist to study end. As the overall time effect was not statistically significant and we did not correct for multiple comparisons, these observations may reflect chance effects. Our study was not designed to assess the mechanisms by which testosterone might exert such effects (if real), but we would speculate in view of the pleiotropic actions of testosterone that it might be mediated by central nervous system responses that might take time to materialize. Of note, there is precedence for a time-limited effect of testosterone treatment. In the T-Trials, the benefit of testosterone treatment on sexual activity was most pronounced during the early phase of the study and waned toward the study end (3); A time-dependent risk of testosterone treatment-related venous thromboembolism has also been reported (20).

In contrast to the effects of testosterone treatment, weight loss and reduced waist circumference were associated with improvements in mental health, irrespective of whether participants received testosterone treatment or placebo. The importance of better baseline HR-QOL and psychosocial function on clinical outcomes is also underscored by our finding that higher baseline HR-QOL and sense of coherence scores correlated with greater decreases in weight over the course of the 2-year RCT. A higher baseline burden of depressive symptomology as reflected by the CES-D score was associated with weight gain. Moreover, higher baseline SF-12 mental scores (reflecting better mental health) were associated with greater odds of participants attending WW.

Collectively, the data support the importance of a holistic treatment approach to men at high risk of diabetes including the optimization of physical and mental health function and extending to the identification and treatment of depressive symptomatology. Conversely, the data also suggest that exposure to lifestyle intervention including weight loss in overweight/obese men is important to optimize HR-QOL and psychosocial function.

Interestingly, in contrast to our hypothesis, there was no interaction between baseline HR-QOL/psychosocial function and the effects of testosterone treatment on glucose outcomes of T4DM. These findings are consistent with a recent mediation analysis from T4DM that found that the testosterone treatment effect was mediated by factors other than HR-QOL and psychosocial function measures, namely by predominantly changes in fat mass (21).

Strengths of this study include its large size and relatively long duration, use of effective testosterone treatment with injections administered by study staff ensuring virtually 100% compliance, use of a relatively large number of distinct HR-QOL and psychosocial function measures using standard questionnaires, and the concomitant lifestyle intervention program. While previous studies have assessed the effects of testosterone treatment on HR-QOL and psychosocial function, our study is unique in that it integrates a well-established and widely used lifestyle intervention (WW) aimed at weight loss within an RCT of testosterone therapy vs placebo. This trial design allowed us to compare the effectiveness of exposure to a lifestyle-based intervention for weight loss to testosterone therapy plus lifestyle intervention on HR-WOL, mood, and psychosocial function. As such, our design allows for addressing the common dilemma of whether to recommend testosterone therapy over and above a lifestyle intervention, providing guidance to clinicians counseling patients on treatment options. Such information has not previously been available as no prior large RCT of testosterone therapy has incorporated a lifestyle program.

Study limitations include the use of self-reported HR-QOL and psychosocial function measures and that some of the analyses conducted were post hoc. Men were selected on the basis of greater risk of diabetes associated with a serum testosterone of <14 nmol/L (by immunoassay) (13) and irrespective of baseline HR-QOL and psychosocial health. Therefore, our findings may not be generalizable to men with pathologic hypogonadism (an exclusion criterion) or to men with a serum testosterone below the reference range for healthy young men and symptoms consistent with androgen deficiency, in whom different effects of testosterone therapy might be expected. Unfortunately, as we did not aim to collect weight data after the study ended, except for a small subset of men (22) for whom weight was self-reported rather than objectively assessed as in the primary trial, we are unable to address whether HR-QOL and psychosocial function had any association with weight change after the RCT ended.

In conclusion, while for men enrolled in the T4DM study testosterone treatment had no effect on most measures of HR-QOL or other measures of psychosocial function, it did improve sense of coherence and subjective social status in a time-limited fashion. Importantly, reductions in body weight and waist circumference occurring during the study irrespective of treatment status (placebo or testosterone) had positive effects on HR-QOL and several indicators of psychosocial status. Moreover, better baseline HR-QOL was associated with larger weight loss in this cohort of overweight/obese men.

Collectively, the findings implicate the importance of a holistic treatment approach to men at high risk of diabetes with a focus on healthy lifestyles to achieve weight loss while at the same time optimizing HR-QOL (eg, addressing reduced physical function, identifying and treating underlying depressive symptomatology) to achieve optimal health outcomes in populations of men akin to those enrolled in T4DM.

## Data Availability

The datasets generated during and/or analyzed during the current study are not publicly available but are available from the corresponding author on reasonable request.

## Clinical Trial Information

Australian New Zealand Clinical Trials Registry. ACTRN12612000287831. https://www.anzctr.org.au/Trial/Registration/TrialReview.aspx? id=362125

## Abbreviations

T4DM: testosterone for diabetes mellitus
